# Dynamic Intraligamentary Stabilization (DIS) for treatment of acute anterior cruciate ligament ruptures: case series experience of the first three years

**DOI:** 10.1186/s12891-015-0484-7

**Published:** 2015-02-13

**Authors:** Philipp Henle, Christoph Röder, Gosia Perler, Sven Heitkemper, Stefan Eggli

**Affiliations:** 1 Sonnenhof Orthopaedic Center, Department of Knee Surgery and Sports Traumatology, Buchserstrasse 30, CH-3006 Bern, Switzerland; 2 Institute for Evaluative Research in Medicine, University of Bern, Stauffacherstrasse 78, CH-3014 Bern, Switzerland

**Keywords:** Anterior cruciate ligament, Dynamic intraligamentary stabilization, ACL suture, Arthroscopic surgery, Sports injury, Knee injury, Microfracturing, Ligamys™

## Abstract

**Background:**

In recent years, the scientific discussion has focused on new strategies to enable a torn anterior cruciate ligament (ACL) to heal into mechanically stable scar tissue. Dynamic intraligamentary stabilization (DIS) was first performed in a pilot study of 10 patients. The purpose of the current study was to evaluate whether DIS would lead to similarly sufficient stability and good clinical function in a larger case series.

**Methods:**

Acute ACL ruptures were treated by using an internal stabilizer, combined with anatomical repositioning of torn bundles and microfracturing to promote self-healing. Clinical assessment (Tegner, Lysholm, IKDC, and visual analogue scale [VAS] for patient satisfaction scores) and assessment of knee laxity was performed at 3, 6, 12, and 24 months. A one-sample design with a non-inferiority margin was chosen to compare the preoperative and postoperative IKDS and Lysholm scores.

**Results:**

278 patients with a 6:4 male to female ratio were included. Average patient age was 31 years. Preoperative mean IKDC, Lysholm, and Tegner scores were 98.8, 99.3, and 5.1 points, respectively. The mean anteroposterior (AP) translation difference from the healthy contralateral knee was 4.7 mm preoperatively. After DIS treatment, the mean 12-month IKDC, Lysholm, and Tegner scores were 93.6, 96.2, and 4.9 points, respectively, and the mean AP translation difference was 2.3 mm. All these outcomes were significantly non-inferior to the preoperative or healthy contralateral values (p < 0.0001). Mean patient satisfaction was 8.8 (VAS 0–10). Eight ACL reruptures occurred and 3 patients reported insufficient subjective stability of the knee at the end of the study period.

**Conclusions:**

Anatomical repositioning, along with DIS and microfracturing, leads to clinically stable healing of the torn ACL in the large majority of patients. Most patients exhibited almost normal knee function, reported excellent satisfaction, and were able to return to their previous levels of sporting activity. Moreover, this strategy resulted in stable healing of all sutured menisci, which could lower the rate of osteoarthritic changes in future. The present findings support the discussion of a new paradigm in ACL treatment based on preservation and self-healing of the torn ligament.

## Background

Optimal treatment after anterior cruciate ligament (ACL) rupture is still intensely debated. A conservative treatment approach shows satisfactory results in patients who place low demands on the knee joint [1-3], but the failure rate remains high in a physically active population [2,3]. Almost every second ACL rupture appears with concomitant injuries, such as unstable tears of the menisci, which have a much lower healing rate when treated conservatively in an unstable environment [4]. Consequently, early secondary injuries to the menisci and cartilage are often found after failed conservative treatment, which may cause rapid degeneration of the knee joint [5].

Current techniques of ACL reconstruction have demonstrated biomechanical, three-dimensional reestablishment of knee joint stability, enabling patients to perform pivoting sports, but compared with conservative treatment decreased degeneration of the knee joint could not be demonstrated [6]. Barrack et al. described a significant loss of knee proprioception with impaired muscular stabilization after ACL rupture [7], and Jerosch et al. showed no significantly better proprioception after ACL transplant compared with the preoperative group [8]. Loss of the ACL’s “proprioceptive envelope” could hence be one explanation for the high incidence of posttraumatic osteoarthritis after ACL injuries, which cannot be overcome by an ACL transplant.

A *restitutio ad integrum* of the ACL would ideally preserve both the neural and the ligamentous stabilizing functions of the ACL. However, spontaneous recovery of the ACL is probably only possible in partial or completely intrasynovial ruptures. The poor healing capacity of the torn ligament—caused by biological factors, the hostile environment of the synovial fluid [9,10], the lack of blood supply [11,12], and the postinjury instability separating the ligament stumps by 5–10 mm—compromises self-healing and the formation of stable scar tissue [13-15]. In addition, the stumps are often dislocated anteromedially and are consequently unable to reattach at the anatomical footprint [16]. Recent studies nonetheless support the potential of biological self-healing of the ruptured ACL leading to a living, proprioceptive structure [17-19].

Dynamic intraligamentary stabilization (DIS) was successfully tested in a biomechanical human cadaver [20] and in a sheep model [21]. It was then clinically applied in a series of 10 physically active individuals [22]. The purpose of the current study was a detailed report on 278 prospectively documented and systematically evaluated DIS patients over a period of three years. We hypothesized that the large majority of them would achieve pre-injury activity levels and be highly satisfied with their treatment outcome.

## Methods

Inclusion criteria: Between February 1, 2011, and January 31, 2014, a total of 278 patients were treated with a dynamic intraligamentary stabilization. Inclusion criteria were as follows: acute ACL injury (time to surgery 21 days or less); closed growth plates; patient not eligible for conservative treatment or not accepting it. Conservative treatment was recommended if all of the following criteria were fulfilled: no more than a 3 mm difference in AP translation when compared with the uninjured contralateral side; no pivoting sports; no meniscal lesions.

Operative technique: The operative technique has been described previously [22]. Briefly, the tibial remnants of the torn ACL are reduced to the femoral footprint by transosseous sutures (anatomical repositioning). After extensive microfracturing at the femoral footprint, the knee is stabilized with a strong polyethylene cord, which is passed on the tibial side behind the tibial footprint, thus preventing the tibial blood and nerve supply from additional damage, and on the femoral side through the anatomical footprint. This cord is brought under tension by a spring-screw implant (Ligamys™, Mathys Ltd Bettlach, Switzerland), which is placed on the anteromedial aspect of the tibia just above the pes anserinus insertion. Thus, the proximal tibia is pulled in a constant posterior drawer position with a force of 50 to 80 N, depending on the weight of the patient. The spring allows a dynamic excursion of 8 mm [23], ensuring a continuous tension of the cord over the entire range of motion, as well as when the polyethylene cord is not placed in an anatomical position.

**Figure 1:**
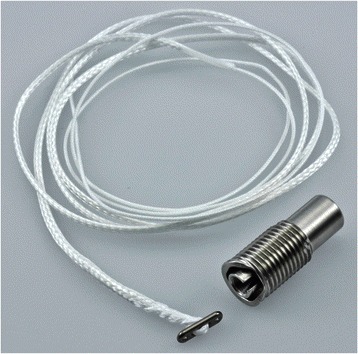
**The Ligamys™implant.** The monobloc with polyethylene cord and endobutton.

In contrast to the pilot series, the surgical technique has been advanced and the necessary surgical instruments improved. The tibial ACL stump is now augmented with up to 5 (average 3) PDS 2–0 sutures. This allows a more accurate reduction of the remaining tissue even in grossly damaged ligaments. The implant and the tensioning device underwent several evolutionary steps, making the implant easier to handle and the applied tension more accurate and reliable. The recent version of the tibial implant is premounted (monobloc system) and the thread is now self-cutting. In addition, the tensioning device now fits in the inner aperture of the implant, leading to a stable connection during the tensioning process (Figure 1).

Clinical evaluation: In accordance with the pilot study protocol of the 10 patients, all patients in the current study were evaluated at 6 weeks and at 3, 6, 12, and 24 months after surgery. The same instruments were used for outcome assessment at each follow-up visit: Tegner, Lysholm, International Knee Documentation Committee (IKDC), and visual analogue scale (VAS) for patient satisfaction (0 = completely dissatisfied, 10 = completely satisfied). Based on the instruments’ outcomes a new combined success definition was applied. Their preoperative scores were assessed as early as possible, but naturally after the trauma. Knee laxity was assessed by measuring anterior translation at 30 degrees flexion with an arthrometer (Rolimeter, Aircast, Neubeuern, Germany) and comparing it with the contralateral knee. All patients were informed that their treatment and follow-up data would be recorded in a scientific database for evidence generation and postmarket surveillance of Ligamys™ and its outcomes, for which they gave their voluntary written informed consent. The study was approved by the Cantonal Ethics Committee of Berne, Switzerland: Ref.-Nr. KEK-BE: 048/09.

**Figure 2:**
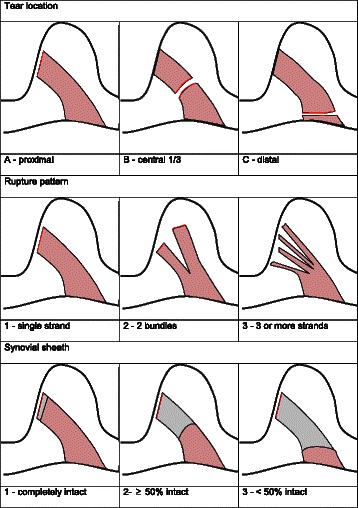
**Anterior cruciate ligament rupture classification.** Classification of ACL ruptures based on the three characteristics rupture location, status of ACL stumps and status of synovial tube.

ACL rupture classification: A 3-digit ACL rupture classification was introduced on the basis of the model of AO classification for long bones [24]. The first digit describes the ACL rupture location: A for proximal third, B for central third, and C for distal third. The second digit is the ACL rupture status: 1 for 1 bundle, 2 for 2 bundles, and 3 for multilacerated. The third describes the ACL synovial tube: 1 for completely intact, 2 for ≥50% intact, and 3 for <50% intact (Figure 2).

Statistics: A one-sample design with a non-inferiority margin was chosen to compare the preoperative and postoperative IKDC and Lysholm scores. Non-inferiority was declared if the lower margin of the one-sided 95% confidence interval for the difference in the means lied above a clinically defined minimum acceptable threshold. For this study, a threshold of 84 percent/points for the IKDC and Lysholm scores, and of < =3 mm compared with the healthy contralateral side for the anterior translation difference was considered clinically acceptable. All analyses were conducted using SAS 9.4 (SAS Institute, Inc., Cary, NC, USA).

## Results

### Characterization of the study population

A total of 278 patients were treated between February 1, 2011, and January 31, 2014. There were 115 women (41%) and 163 men (59%). Mean (SD) age was 32.4 (11.4) years (range 18–63 years), and mean (SD) body mass index was 24.1 (3.29) (range 16–35.4). The right:left knee ratio was 119:159 (left 57.19%). The mean accident-surgery interval was 18.0 (29.93) days. In 240 patients (86%), there was no preexisting lesion of the injured knee. The other 38 patients had an average of 2 intraoperatively observed preexisting lesions. The most frequent ones were abrasions of the medial femoral cartilage 25 patients, of the lateral one in 11; 8 patients showed degenerative lesions of the medial meniscus, 2 of the lateral one. 173 (62%) patients showed additional trauma related lesions of the injured knee. 127 patients (46%) had 1 additional lesion, 42 patients (15%) had 2 additional lesions, 3 patients (1%) had 3 additional lesions, and 1 patient (0.4%) had 4 additional lesions. 152 patients (55%) had an additional lesion of one or both menisci. In 98 patients (36%), we immediately sutured the torn menisci and in another 25 patients (9%), we partially resected them. In 29 patients, the meniscal lesions were superficial and stable and therefore not suitable for suturing. The mean (SD) operation time was 53.6 (14.2) minutes (range 30–120 minutes), and the mean (SD) hospitalization time was 1.9 (0.9) days (range 1–5 days).

### Rupture classification

Applying the newly introduced rupture classification, the 3 most frequent rupture classes were an A.2.2 rupture (proximal third, 2-bundle rupture, ≥50% of synovial tube intact) in 58 patients (21%), followed by an A.1.1 rupture (proximal third, 1-bundle rupture, synovial tube intact) in 56 patients (20%), and an A.1.2 rupture (proximal third, 1-bundle rupture, ≥50% of synovial tube intact) in 36 patients (13%). Table 1 shows the overall distribution of the 3 different rupture characteristics and their occurrence.

**Table 1:** **ACL rupture characteristics and percentage of their occurrence in patient groups**

Location	A (proximal third)	B (middle third)	C (distal third)
	73.4%	26.2%	0.4%
Status	1 (1 bundle)	2 (2 bundles)	3 (multilacerated)
	46.8%	36.7%	16.5%
Synovial tube	1 (intact)	2 (≥50% intact)	3 (<50% intact)
	24.8%	50.4%	24.8%

### Available postoperative data

There were 148 3-month follow-ups; 197 6-month follow-ups; 204 12-month follow-ups; 69 24-monthfollow-ups; and 2 follow-ups in other intervals. The mean interval of the last follow-up was 14 months; the maximum interval was 3 years.

### Patient reported outcome measures and clinical evaluation

The mean (SD) Lysholm score was 99.3 (2.3) points (range 81–100, N = 277) before injury, 91.8 (6.9) points (range 68–100, N = 148) after 3 months, and 97 (5.0) points (range 75–100, N = 69) after 24 months. The mean (SD) IKDC score was 98.8% (6.4) (range 87-100%, N = 277) before injury, reaching 83.2% (9.6) (range 56-100%, N = 148) after 3 months and 94.8% (6.5) (range 74-100%, N = 69) after 24 months.

By improving from a mean (SD) of 3.7 (1) points after 3 months (range 1–10 N = 148) to a mean 5.1 (1.4) points after 24 months (range 3–10 N = 69), the group’s mean Tegner score nearly reached the pre-injury level of 5.1 (1.4) points (range 3–10). The mean (SD) patient satisfaction was 8.1 (1.5) (range 4–10, N = 148) after 3 months and 8.9 (1.3) (range 5–10, N = 69) after 24 months. Before surgery, the mean (SD) anterior translation difference between the injured and the healthy contralateral knee was 4.7 (2.0) mm (range 0–11 mm). After 3 months, it was 0.8 (1.4) mm (range −3 to 5 mm) and after 24 months, it was 2.3 (1.7) mm (range −2 to 6 mm) (Table 2).

**Table 2:** **Pre- and postoperative outcome scores, ap translation differences and patient satisfaction**

Scores/tests	Before injury	3 months postoperative	6 months postoperative	12 months postoperative	24 months postoperative
	(N = 278)	(N = 128)	(N = 171)	(N = 176)	(N = 62)
Lysholm	99.3 (2.3)	91.8 (6.9)	95.13 (6.2)	96.07 (6.46)	97 (5)
2Y (N = 62)	99.4 (2.12)	95.4 (4.7)	95.06 (8.0)	95.7 (7.0)	97.4 (4.6)
IKDC (%)	98.8 (6.4)	83.2 (9.6)	90.4 (8.0)	93.5 (8.0)	94.8 (6.5)
2Y	99.3 (2.1)	89.0 (5.7)	90.9 (8.8)	92.8 (8.9)	95.4 (6.1)
Tegner	5.1 (1.4)	3.7 (1)	5.0 (1.4)	5.1 (1.4)	5.1 (1.4)
2Y	5.2 (1.3)	4.2 (1.1)	4.4 (1.2)	5.0 (1.5)	5.1 (1.4)
Delta Lachmann contralateral knee (mm)	4.4 (2.3)	0.5 (1.9)	1.0 (1.8)	1.4 (1.7)	2.0 (1.6)
2Y	4.2 (2.19)	0.2 (2.2)	1.0 (1.5)	1.4 (1.6)	1.9 (2.0)
Delta Lachmann healthy contralateral knee	4.7 (2)	0.8 (1.4)	1.0 (1.7)	1.5 (1.7)	2.3 (1.7)
2Y	4.4 (1.9)	0.5 (1.9)	1.1 (1.3)	1.5 (1.5)	2.1 (1.7)
Patient satisfaction (VAS)		8.1 (1.5)	8.8 (1.2)	8.9 (1.3)	8.9 (1.3)
2Y		9.3 (0.9)	9.0 (1.3)	8.9 (1.3)	9.0 (1.3)

Mean scores and standard deviations before and after surgery and mean anterior translation differences with standard deviations for all patients and those with a contralateral knee without previous ligamentous injury. Results of the 62 patients with 2-years follow-up are listed as 2Y.

### Combined success

When a combined success definition was applied (AP translation difference ≤3 mm, Lysholm score >84 points [25], IKDC score >84% [26]), 77.2% (N = 210 of patients) fulfilled all 3 criteria at the last follow-up. The distribution of the individual success criteria was as follows: AP translation ≤3 mm, 86.8%; Lysholm score >84 points, 95.6%; and IKDC score >84%, 87.6%. For the 60 patients (22.8%) with a satisfactory outcome, the distribution of the individual success criteria was as follows: AP translation ≤3 mm, 41.9%; Lysholm score >84 points, 80.7%; and IKDC score >84%, 45.2%.

### Statistical assessment

The non-inferiority test revealed lower confidence limits of 95.2 points for Lysholm and of 91.6% for the IKDC scores. The lower confidence interval of the anterior translation difference to the healthy contralateral side was 1.3 mm. Hence all mean outcome scores and values were above the predefined thresholds (p < 0.0001 for all three outcomes).

### Return to work

There was a mean (SD) of 79.9 (29.4) days until 100% return to work (range 4–120 days) for patients who did heavy physical labor; 50.6 (39.4) days (range 1–191 days) for patients who did light physical labor; and 25.1 (19.0) days (range 0–100 days) for patients with a sitting occupation.

### Intra- and postoperative complications

Three intraoperative complications occurred in 3 patients (1.1%): 1 perforation of the endo button into the femur, 1 second screw tunnel because of insufficient anterior coverage, and 1 Kirschner wire breakage. All situations could be solved without additional damage to the knee. There were 8 complications in 8 patients until hospital discharge (2.9%): 5 hematomas and 3 others (pneumonia, severe obstipation, wound dehiscence).

### Treatment failure rate

**Figure 3:**
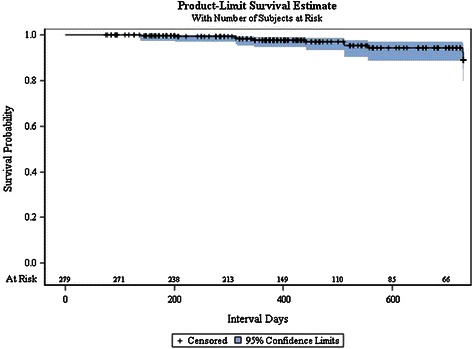
**Survival curve of dynamic intraligamentary stabilization treatment.** Rerupture (N = 8) or mechanical insufficiency (N = 3) are defined as the endpoints of survival.

Eight reruptures of the ACL occurred at an average of 338 days after surgery (range 106–740 days). None of the cases had had any intra- or postoperative complications. All were treated with a ligament graft. There were 3 reported mechanical insufficiencies of the ACL (giving way). Figure 3 shows the survival curve of Ligamys™ implants with rerupture or mechanical insufficiency as the endpoint.

### Implant (monobloc) removal

**Figure 4:**
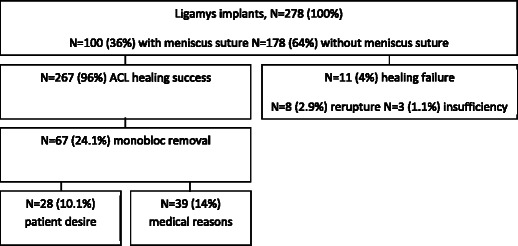
**Total number of Ligamys™implantations and further course of treatment.** Faith algorithm of ACL ruptures, DIS treatment success and failures, as well as monobloc removals and reasons.

The implant was removed postoperatively in 67 (24.1%) patients, of whom 28 (10.1%) asked for implant removal without any clinical need. In the other cases, the implant was removed because of infection (2 patients, 0.7%), pain (14 patients, 5.0%), or joint stiffness (17 patients, 6.1%); in the remaining cases (6 patients, 2.2%), the implant was removed during interventions for meniscal rerupture, a free-floating body in the joint, or crepitations (Figure 4).

## Discussion

The most important findings of the current study are that its results corroborate those of the pilot group [22]. This group had consisted of 10 highly selected and motivated young and physically fit individuals, with a ratio of men to women of 8:2 and an average age of 25 years. The current patient sample is more representative regarding the future and more widespread use of DIS. With a ratio of men to women of 6:4 and an average age of 31 years, the patient population has shifted from a clear domination of young men with an intense level of recreational sports activity to a more normally distributed sample of physically active individuals. The short and mid- term results of DIS, however, remain excellent.

The preoperative Tegner activity scale between the pilot group and the current population changed from 6 to 5, reflecting a more sportive patient group in the pilot study. As could be expected with an increasing number of treated patients, the range of results became more widespread, which we consider to be a trend towards normalization rather than one towards worsening. At 6 months after surgery, the median IKDC and Lysholm scores were both above 90, and after 12 months, they had returned to levels of close to 100 with the same Tegner scale as before the ACL rupture. However, there were also some 6-month scores of around 50 points for the Lysholm and IKDC scales, which remained low at 12 months. Nevertheless, overall patient satisfaction was already very high at 6 months, and it further increased with longer follow-up intervals. The 24-months results, despite being at a desirably high level, only represent a 25% follow-up rate, with more patient examinations expected in future.

Our main conclusion from the results of the first 10 patients—namely, that DIS of the knee with a freshly ruptured ACL, in combination with anatomical repositioning and biological improvement of the healing environment, can lead to a biomechanically stable ACL with good functional scores and high patient satisfaction—can now be generalized to a much higher degree. A bias towards optimal results was introduced by the motivation and physical fitness of our first 10 patients. However, the desired functional outcomes in the present 278 cases, an AP side-to-side difference ≤3 mm, a Lysholm score >84 points, and an IKDC score >84%, were reached in 86.8%, 95.6%, and 87.6% of patients, respectively, at the last follow-up. In applying our combined success definition, we found that 77.2% presented an excellent outcome.

It was surprisingly difficult to compare outcomes with other authors. The initial conservative approach with delayed surgery in case of unsatisfactory results leads to pre-surgery instead of pre-injury reporting of activity levels and scores. Since DIS must be performed within few weeks after ACL rupture, we routinely assess patients’ pre-injury activity levels, even if with a retrospective perspective. Streich et al. [27] compared outcomes of double bundle versus single bundle semitendinosus grafts in male athletes and reported pre-injury Tegner activity levels of about 8, and of 7 two years after surgery without any significant group differences. IKDC and Lysholm scores were both around 90 at 2 years after surgery, again without significant intergroup differences. Pre-injury values were not reported. Park et al. compared hamstring based single bundle versus double bundle ACL repair in more normal patient groups with average ages of 28 and 29 years and found Tegner scores of about 5.5 in both groups at 2 years after surgery [28]. Frobell RB et al. compared structured rehabilitation with early ACL reconstruction versus structured rehabilitation with optional delayed ACL reconstruction in an RCT design and measured 2 years-median Tegner activity scores of 6.5 in the early intervention group and of 5 in the optional delayed intervention group. Both groups were about 26 years of average age [3]. Finally a meta-analysis of Biau et al. reported that only about 40% of patients made a full recovery after ACL reconstruction, with only 33% having a normal IKDC score after a semitendinosus transplant and 41% having a normal IKDC after a BTB (ligamentum patellae) transplant. Thus, more than 60% of patients (708 of 1,125 for the two reconstruction groups) did not fully recover (final overall IKDC score class A) after reconstruction [29].

Our findings add further evidence to the paradigm shift that a torn ACL has sufficient healing capacity. We have already presented results of other studies indicating that the injured ACL can produce a stable scar under certain circumstances. Sutures with tissue augmentation [15], placement of undifferentiated stem cells into the rupture zone [30], conservative treatment with an extension block soft brace without anterior stabilization [31], or primary sutures in combination with bone marrow stimulation [32] all lead to scarring or healing of the ACL with consequently improved stability and function in a considerable number of patients.

The essential prerequisites for ACL healing are mainly the same as they are for all biological tissues: stability and integrity of a healing environment. The increased AP translation of the knee with a ruptured ACL leads to a constant disconnection of the 2 ACL stumps and thereby compromises healing by instability [33]. A method of external bracing in posterior translation has already shown success in healing many ACLs, but its discomfort hampers its wider application [31,34]. We have developed a technique for internal dynamic stabilization of the knee by using a screw-spring mechanism that acts as a dynamic internal fixator. It pushes the knee into a maximum posterior translation in any degree of flexion and is also functional when the intraligamentary thread is not placed in an isometric position. This is the crucial point of the technique because the spring allows for a non-anatomical placement, mainly on the tibial side, thus preventing additional trauma to the tibial blood and nerve supply. All previous rigid systems had to be placed isometrically and therefore had to go through the tibial anatomical footprint, which adds additional damage to the biological integrity of the ligament. DIS was first applied in a sheep model and provided sufficient stability to enable biomechanically stable healing of the ACL [21]. Addressing the crucial biological aspects in the pilot series, we made use of the work of Mastrangelo, Murray, and Zumstein [18,35-38] to increase ACL healing capacity by introducing a collagen-platelet composite and solid scaffolds for long-term delivery of growth factors, especially leukocyte- and platelet-rich fibrin (L-PRF). We added Steadman’s microfracturing technique [30] as a further measure for improving the biological healing capacity of the ACL. In the present larger patient group, we abandoned the L-PRF preparation because of recently published negative results about ligament healing [39,40]. Our clinical data confirmed that this change of strategy did not change the clinical and mechanical results.

AP translation measurements at 6–12 months after surgery are only a surrogate measure for the reestablished static stability of the knee joint, but its correlation with patient satisfaction scores was reported to be poor, whereas proprioception is a key aspect in measuring the overall outcome of an ACL reconstruction [41]. Patients in the present study reported satisfaction of 9 out of 10 on the VAS after 1 year, indicating that healing of the ACL tissue may restore not only the 3-dimensional stability of the knee, but also the physiological proprioceptive envelope without adding additional trauma to the knee such as harvesting a donor graft. We attribute the excellent clinical results of the DIS technique to the restored stability of the knee, but even more to the preservation of the ACL tissue, which may allow for the restoration of physiological proprioception. This restoration may also positively affect the rates of late osteoarthritis, which are controversially stated to be higher in patients after surgical ACL grafting compared with conservative treatment [6,42,43] and which may be attributable to loss of proprioception after complete removal of the ACL [1,44,45], as well as to insufficient restoration of the 3-dimensional stability of the knee [46-48]. However, these osteoarthritis rates cannot yet be measured or predicted.

The failure rate of DIS, either as a rerupture of the ligament or a clinically unstable knee, was 3.95% until the end of the study period. Following up on 612 patients, Salmon et al. [49] found a 6.4% rerupture rate at 5 years after an ACL patellar or hamstring tendon graft. Mariscalco et al. [50] found a 5.3% revision rate in 263 patients 2 years after a hamstring tendon graft. The failure rate of DIS is still lower than that reported for an ACL graft, but the 2-year follow-up rate must be completed for more conclusive comparisons. On the other hand, a revision situation after DIS is almost a normal ACL repair surgery because all ligaments are still available for reconstruction – a normal ACL repair with a tendon graft remains as a salvage procedure if the healing procedure fails.

One of the major drawbacks in conservative treatment is the neglect of the concomitant injuries after an ACL injury, mainly the meniscal tears. A missed unstable meniscus, combined with knee instability, will destroy the meniscal tissue and consequently end in surgical removal [51,52]. In the current series, we performed an immediate meniscal suture combined with DIS in 98 patients (36%), thereby creating an ideal environment for meniscal healing. In 29 patients, the tear was only superficial and therefore mechanically stable. Only 1 patient had to undergo revision surgery with an additional successful suture. That means that in 100% of our patients, unstable meniscal tears could be preserved by immediate suturing and stabilization of the knee. According to the latest literature, this could be another factor to decrease the rate of arthritic changes in the future [53-55].

Limitations of the study: The current study has weaknesses that need to be considered. Being a case series, the study can only generate level-four-evidence. We had initially designed a randomized trial for the first ten patients, but a 100% cross over rate of patients allocated to conventional ACL reconstruction made it impossible to conduct the trial. Despite reaching a 2-years maximum follow-up in 22% of reported cases, the average follow-up time is only 14 months and the topic of ACL repair or reconstruction and their long-term outcomes needs much longer follow-up intervals. Further, it is unclear when the internal brace fails and what the consequences for ap-translation of the affected knee are. First analyses revealed a minimal translation increase if the monobloc was removed, but without any clinical or functional consequences. Finally, restoration of knee proprioception after DIS is a clinical hypothesis that cannot yet be proven.

## Conclusions

Anatomical repositioning, along with DIS and microfracturing of the notch, leads to clinically stable healing of the torn ACL in the large majority of patients. Most patients exhibited almost normal knee function, reported excellent satisfaction, and were able to return to their previous levels of sporting activity. Moreover, this strategy resulted in stable healing of all sutured menisci, which could lower the rate of osteoarthritic changes in future. The present findings support the discussion of a new paradigm in ACL treatment based on preservation and self-healing of the torn ligament.
